# Disinhibiting dendritic cell-natural killer cell cooperation against metastasis

**DOI:** 10.1007/s44466-026-00060-2

**Published:** 2026-09-23

**Authors:** Oliver Kepp, Guido Kroemer

**Affiliations:** 1 University of Paris-Saclay, INSERM US23, Gustave Roussy, Villejuif, France; 2 Université Paris Cité, Sorbonne Université, INSERM UMRS1138, Centre de Recherche des Cordeliers, Paris, France; 3 Department of Biology, Institut du Cancer Paris CARPEM, Hôpital Européen Georges Pompidou, AP-HP, Paris, France

**Keywords:** Annexin A1, Cancer immunosurveillance, Dendritic cells, Formyl peptide receptor 1, Natural killer cells

## Abstract

Metastatic tumors evade not only adaptive but also innate immune control. In a recent paper published in *China Science Life Sciences*, Jin and colleagues identify EID1 as a transcriptional and functional brake shared by dendritic cells (DCs) and natural killer (NK) cells. Genetic or nanoparticle-mediated EID1 inhibition strengthens DC-NK apposition, augments FPR1-dependent type I and II interferon responses through a mechanism involving FPR1 and potentially NK cell-derived ANXA1, and suppresses experimental metastatic colonization/outgrowth. The work connects a transcriptional-corepressor to innate immunosurveillance and suggests that the previously described ANXA1-FPR1 danger-sensing module may operate not only between dying cancer cells and DCs in primary sites, but also among NK cells and DCs at metastatic lesions.

## Main text

Impaired immunosurveillance and immune evasion facilitate carcinogenesis and tumor progression. Similarly, metastasis is not merely the late consequence of cancer cell evolution but also the product of immune failure. Disseminated cells must survive in the circulation, colonize foreign tissues and escape immune populations that are particularly proficient at eliminating isolated or stressed targets. Among these, natural killer (NK) cells provide rapid cytotoxicity, whereas dendritic cells (DCs) sense tissue perturbation, organize local inflammation and bridge innate effector cells to adaptive T lymphocyte-mediated immunity. DC-NK cooperation has long been recognized as reciprocal: DC-derived type I interferons (IFNs) and interleukins (ILs) activate NK cells, while NK-derived IFN-γ and chemokines mature and recruit DCs [1, 2]. What has remained less clear is whether a shared intracellular brake can simultaneously disable both partners in metastatic sites.

Jin and colleagues now identify E1A-like inhibitor of differentiation 1 (EID1) as such a brake [3]. EID1 is a short-lived transcriptional corepressor that binds CBP/P300 and couples cell-cycle exit to differentiation [4]. Mining single-cell transcriptomes from 120 patients with liver cancer, the authors found *EID1* to be the only surveyed histone-acetylation regulator increased in both malignant cells and effector lymphocytes in metastatic, compared with non-metastatic, lesions [3]. This observation is intriguing, although not causal: metastatic samples may differ in anatomy, cellular composition and treatment history, and EID1 messenger RNA does not necessarily report the abundance or activity of this rapidly degraded protein.

The functional evidence is considerably stronger. Whole-body *Eid1* deletion reduced pulmonary colonization by intravenously injected B16F10 melanoma and EO771 mammary carcinoma cells, and limited multifocal hepatic growth of Hepa1-6 tumors. Consequently, it prolonged survival of mice with metastatic diseases. NK-cell depletion erased this protection, whereas depletion of CD4^+^ or CD8^+^ T cells did not. Moreover, *Eid1*-deficient NK cells displayed increased proliferation, survival, NKG2D expression, degranulation and IFN-γ production, and outperformed wild-type NK cells after adoptive transfer. NK-restricted *Eid1* deletion reproduced part, but not all, of the phenotype, arguing that NK cell-intrinsic reprogramming is necessary but profits from a permissive multicellular environment [3].

**Fig. 1:**
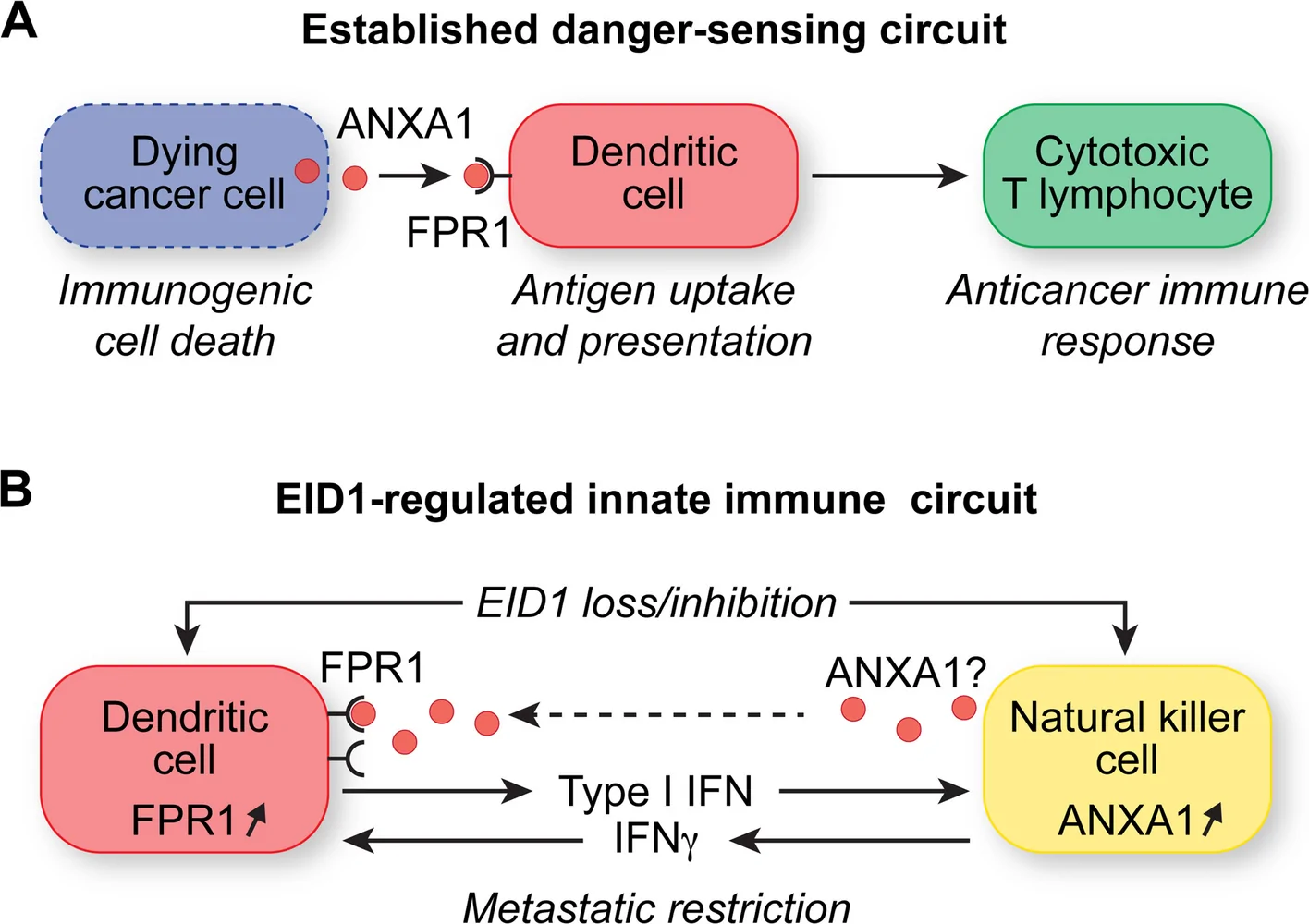
EID1 restrains a proposed ANXA1-FPR1-dependent DC-NK circuit. The model depicts EID1 loss which increases FPR1 in DCs and ANXA1 in NK cells, promotes cellular apposition and amplifies DC-derived type I IFNs and NK-derived IFN-γ, thereby restricting metastatic outgrowth. Prior work established a parallel input in which ANXA1 released from dying cancer cells activates FPR1 on DCs to support corpse sensing, antigen uptake and T-cell priming. The proposed NK-to-DC ANXA1-FPR1 connection remains hypothetical and requires lineage-specific genetic epistasis

DCs constitute the other arm of this environment. *Eid1* deficiency increased DC accumulation and shortened DC-NK distances within pulmonary metastases as detectable by sophisticated multicolor immunofluorescence and image analysis. Direct DC-NK conjugates were more frequent, and *Eid1*-deficient DCs enhanced NK cell survival, cytotoxicity and IFN-γ production in coculture. DC- or myeloid-restricted *Eid1* deletion also reduced metastasis, albeit less efficiently than systemic deletion. Surprisingly, neither IL-1 nor IL-15 blockade eliminated protection. Instead, transcriptomic ligand-receptor inference nominated annexin A1 (ANXA1) and formyl peptide receptor 1 (FPR1), while pharmacological FPR1 inhibition curtailed DC and NK accumulation, IFN-γ production and metastatic control. *Eid1* loss also amplified type I IFN bioactivity, and neutralizing IFNAR1 or IFN-γ compromised protection. Thus, EID1 appears to restrain a self-reinforcing circuit in which DC-derived type I IFNs and NK-derived IFN-γ turn cellular proximity into metastatic resistance (Fig. 1).

Conceptually, the most interesting feature of the work is its distributed causality. Neither NK-restricted nor DC/myeloid-restricted *Eid1* deletion matched the systemic knockout, suggesting that EID1 does not control one dominant effector but imposes parallel constraints on several cooperating compartments. This distinguishes EID1 from a conventional immune checkpoint expressed by a single lineage. Notably, EID1 was highest in malignant cells in the human dataset, yet the experiments do not separate tumor-cell-intrinsic effects on proliferation or stemness from immune-cell-intrinsic effects on surveillance. Reciprocal transplantation or inducible, compartment-specific silencing would be needed to determine whether malignant and immune EID1 are additive, sequential or mechanistically independent [3]. With further exploration on this notion, the therapeutic potential of EID1-targeted interventions can be unravelled.

Therapeutically, infusion of *Eid1*-deficient NK cells mediated superior anti-metastatic benefits as compared to *Eid1*-proficient counterparts. Alternatively, the authors packaged *Eid1*-directed small interfering RNA in redox-responsive nanoparticles. Repeated systemic administration lowered *Eid1* expression and boosted *Fpr1* transcription in metastatic tissue, expanded activated intratumoral NK cells, eventually reducing mammary carcinoma lung lesions as well as multifocal hepatic tumors. This is an important proof of concept because EID1 lacks an obvious catalytic pocket. Yet it is not a pharmacological endpoint: biodistribution across tumor, endothelial and immune compartments remains unresolved; delayed treatment of established metastases was not tested; and repeated dosing was not accompanied by the toxicology, cytokine profiling or anti-infective assessments required for a strategy designed to systemically unleash IFNs [3].

Moreover, increased *FPR1* expression is proposed as a pharmacodynamic marker, but whole-tissue *FPR1* cannot identify the responding compartment and may reflect altered leukocyte abundance rather than transcriptional de-repression within individual cells. A useful biomarker must therefore be cell-resolved and linked to target engagement, not simply associated with an inflamed lesion. The same caution applies to the survival analyses: high *EID1* and low *FPR1* in bulk or computationally annotated tumor cells cannot validate a mechanism assigned to DCs and NK cells.

This network logic nevertheless provides a rational framework for combining EID1 inhibition with therapies that generate immunogenic tumor debris, recruit cDC1s or amplify NK-cell fitness.

The ANXA1-FPR1 connection gives this study a particularly provocative historical resonance. During immunogenic cancer cell death, ANXA1 released by dying malignant cells functions as a danger signal for FPR1 expressed by DCs. This interaction guides DCs toward corpses, favors their juxtaposition with dying cells and enables antigen uptake, presentation and downstream cytotoxic T-cell responses [5]. Accordingly, loss of host *Fpr1* compromises the efficacy of anthracyclines or oxaliplatin in mice, while the loss-of-function *Fpr1* variant rs867228 is associated with inferior outcomes after adjuvant chemotherapy in breast cancer and chemoradiotherapy in patients with locally advanced rectal cancer [5, 6].

FPR1 also contributes to constitutive, treatment-independent immunosurveillance. The rs867228 allele, carried by approximately one-third of humans across different ethnicities, has been associated with earlier diagnosis of luminal B breast, colorectal, esophageal and head-and-neck carcinomas [7, 8], and *Fpr1* deficiency accelerates inflammation-associated intestinal oncogenesis in mice [9]. These observations have generally been interpreted through defective sensing of ANXA1 emitted by dying or stressed cancer cells [5].

The new work suggests a complementary hypothesis: ANXA1 supplied by NK cells may stimulate FPR1 on DCs, thereby sustaining a DC-NK interferon circuit before abundant tumor-cell death has occurred. If correct, the common FPR1 loss-of-function variant would interrupt two immunosurveillance inputs converging on the same DC receptor: a cancer cell-to-DC signal that supports antigen capture, and an NK cell-to-DC signal that reinforces innate control of dissemination. This dual defect could help explain both earlier cancer manifestation and reduced therapeutic responses [3].

For now, this is a hypothesis, not a demonstrated cellular topology. The study by Jin et al. [3] measures *Fpr1* and *Anxa1* transcripts and uses computational interaction inference, but does not genetically delete *Anxa1* in NK cells, delete *Fpr1* selectively in DCs, quantify extracellular NK-derived ANXA1 or visualize ligand-receptor engagement. Systemic FPR1 antagonism establishes FPR1 dependence, not the identity or direction of its ligand. ANXA1 can also signal through other formyl peptide receptors and exert context-dependent pro-resolving effects. The decisive experiment is therefore a genetic epistasis matrix combining NK-specific *Anxa1* loss with DC-specific *Fpr1* loss in *Eid1*-deficient hosts.

Several additional limitations must be addressed to liberate the path to clinical translation. Intravenous B16F10 and EO771 inoculation measures pulmonary seeding and outgrowth rather than the complete metastatic cascade; the Hepa1-6 model similarly bypasses spontaneous dissemination. Cre-based recombination driven by *Ncr1*-, *Itgax*- and *LysM*-promoters is informative but not perfectly lineage- or subset-restricted, and the study does not establish which conventional DC subset mediates protection. Nor is EID1 shown to occupy or epigenetically silence the *Fpr1* locus. Indeed, P300 inhibition failed to reverse the phenotype, leaving the direct transcriptional mechanism unresolved. Finally, the human evidence remains correlative: *EID1* expression and survival associations do not demonstrate an operational EID1-ANXA1-FPR1 circuit in human metastatic DCs and NK cells [3].

These limitations define an unusually productive experimental agenda. Adult-onset and temporally controlled *Eid1* silencing should be tested against spontaneous and established metastases, followed by chromatin mapping, cell-resolved nanoparticle tracing and validation in human NK-DC cocultures stratified by rs867228. FPR1 genotype may ultimately function as a companion biomarker: *EID1* inhibition should depend on an intact DC receptor, whereas p38α inhibition can restore defective antitumor functions in *Fpr1*-mutant cDC1s in preclinical systems [10]. Thus, EID1 blockade and FPR1-corrective therapy might be complementary rather than competing strategies. Although Jin and colleagues have not yet delivered a metastasis drug, they have uncovered something more foundational: a transcription-associated immune break that restrains an innate immune alliance, and a plausible second life for ANXA1-FPR1 signaling in cancer immunosurveillance.

## Data Availability

No datasets were generated or analysed during the current study.
